# The Changing Patterns of Consumers’ Behavior in China: A Comparison during and after the COVID-19 Pandemic

**DOI:** 10.3390/ijerph18052447

**Published:** 2021-03-02

**Authors:** Xiaoling Yuan, Caijuan Li, Kai Zhao, Xiaoyu Xu

**Affiliations:** School of Economics and Finance, Xi’an Jiaotong University, Xi’an 710061, China; xiaoling@mail.xjtu.edu.cn (X.Y.); lcj12342000@stu.xjtu.edu.cn (C.L.)

**Keywords:** COVID-19, lock down, socio-economic cost, living habits

## Abstract

This paper has an opportunity to collect questionnaire-based data regarding respondents’ life choices in China at the peak of COVID-19 outbreak (i.e., around 9–11 March 2020) and in a relatively stable period where the national pandemic was over and the lockdown policy was halted (i.e., around 25–30 March 2020). Comparing respondents’ answers about their most fundamental aspects of life during and after the pandemic, including income level, expenditure structure and level, purchase method, study method, food price and quality, and dining habit, both the descriptive and econometric models reveal that Chinese consumers’ life patterns were not significantly changed. These findings may imply a “new normal” where consumers stick to their new living habits that were forged during the pandemic. Therefore, policy makers have to envisage such an implicative socio-economic change (cost) brought by the implementation of a lock down policy in a long run, in addition to direct and explicit economic losses. However, improving food quality and controlling food price appear to be the strong and stable safety signals to reassure consumers in this complicated environment.

## 1. Introduction

The COVID-19 pandemic so far is the most serious global public health event in the 21st century [1]; it has posed drastic threats not only to public health, but also to various essential aspects of human society and the global economy [2]. As a result, the unprecedented scale and severity of such a pandemic have aroused increasing interest in various research domains. First, attention was focused in particular on understanding this virus’ pathogenic mechanism [3,4], clinical characteristics and transmission route [5], as its presence has posed significant challenges to global safety in public health. Compared to SARS, COVID-19 is much more infectious. Early stage patients may only have mild symptoms but with large amount of virus in their upper respiratory tracts, and droplets and aerosols are also viral vectors which let the virus be bioactive outside the body for a long period of time. It is evident the survival time of COVID-19 on hard and plastic surfaces is at least up to three days [6] and even longer in an environment with low temperatures [7], thus infection control measures are necessary [8] such as pandemic forecasts [9] or population mobility monitoring [10].

Another thread of literature, from a different perspective, attempted to develop antiretroviral therapies. It is therefore not surprising that significant progress has been made towards drug and vaccine development [11,12]. For instance, the mRNA-1273 vaccine, which is generated by encoding the prefusion-stabilized spike protein of SARS-CoV-2, has been widely proved to be effective in neutralizing SARS-CoV-2 [13], alternatively, other scholars have used the inactivated vaccine method [14].

The pandemic is also considered as a socio-economic crisis, and a cause of environmental changes. On one hand, even though interventions such as highly restrictive social distancing [15], closing public spaces [16] or even a complete lockdown have played significant roles in controlling the spread of COVID-19 [17], there have been negative economic and social implications [18]. These unusual countermeasures, to a certain degree, have affected people’s mental and physical well-being in many ways [19], causing physical [20] and mental health damages [21] or real income decline and unemployment growth [22,23]. On the other hand, human activities were sharply decreased due to the partial-to-total lockdown, thus the normal negative effects of anthropogenic activities appear to be had been mitigated. For instance, Lal et al. [24] have stated that it was an evident global reduction in the levels of NO_2_ and CO during the pandemic.

Yet despite the great effort has been made a range of aspects associated with the presence of COVID-19, existing studies in socio-economic disciplines have not provided sufficient empirical evidences to understand how such an external shock can affect the quality of life. Even though several studies, to a certain degree, have showed that people are more likely to be affect by socio and economic aspects than psychological and physical ones during this pandemic [25], attention was mainly focused on the perspective of people’s mental well-being [26] and specific aspects of their shopping behaviors [27]. Thus, there is a need to develop a theoretical framework that can deepen understanding of the multifaceted phenomenon of the socio-economic cost of public health policies concerning COVID-19.

With this in mind, the study mainly focuses on how the presence of COVID-19 affected people’s perceptions towards life choices in a broader context of socio-economic development and in particular we sought answers to the following questions: RQ (1) how a lockdown policy, as the most notable public health intervention in China, affects people’s perceptions of life choices; RQ (2) how people’s changing perceptions with respect to life choices in turn affect their attitudes towards the efficiency of such a policy. In doing so, this paper describes two waves of surveys among the Chinese population with similar demographic features across regions. The data convenience allows us to separately obtain useful information about the patterns of consumption structure and living habit at the peak of COVID-19 outbreak (i.e., around 9–11 March 2020) and in a relatively stable period where the national pandemic was over and the “lockdown” policy was halted (i.e., around 25–30 March 2020). By integrating the results in these two periods it is expected that more insights will be gained reconcile the inconsistency between public health policy implenetation and actual socio-economic outcomes.

This study contributes to the literature in several ways. First, this study is one of the few studies looking into the overall socio-economic impact of COVID-19 on the life patterns of Chinese population. We link the discussion with a more indirect and implicative socio-economic cost (i.e., behavioral and perceptual changes) of the lock-down policy, widening the stream of academic work on the socio-economic cost of COVID-19 and associated public health policies. Second, this study also introduces a measurement that integrates people’s subjective estimations (i.e., the degree of satisfaction towards the efficiency of a lockdown policy) with their changing patterns of perceptions and behaviors. Third, this study is based on a “during and after” analytical framework, compared to most of the existing studies where only a “before and during” scenario is feasible.

The remainder of this article is structured as follows: The next section details the methods used in collecting the data and our econometric model specifications. Section 3 confirms the quality of the data. In Section 4, both comparative and econometric analyses are conducted in response to RQ 1 and 2. Section 4 discusses the results and Section 5 concludes this work.

## 2. Research Design

### 2.1. Stage 1: Sampling Method

Participants were recruited across all provinces in China excluding Hong Kong, Macao and Taiwan. Using a completely anonymous and voluntary question link that was sent to participants’ cell phones, the basic principle of random sampling can be guaranteed, as the coverage rate of cell phones is far higher than that of internet users [28] in China. It has to be admitted that in some remote areas, even cell phone use is not yet widespread, but local residents in that case are also much more likely to be a poor population whose lifestyles are far from a model style and the pandemic thus there is no basis to discuss aspects of their lifestyles such as shopping habits, online education, etc. Moreover, because outside activities were constrained, an online survey also appears to be the only accessible channel to reach potential respondents under this special situation.

### 2.2. Stage 2: Questionnaire Design and Polit Test

As research on COVID-19 is a relatively new domain, there is no a well-accepted “convention” to follow for designing questionnaire. Therefore this study refers to the design philosophy of Wendy, et al. [29] and Zervides, et al. [30], and constructs four behavioural and perceptual dimensions that represent people’s lifesyles and associated perceptions towards the pandemic and policy-makings. An initial questionnaire design including 26 measurement items was tested on 100 vlunteers who provided feedback on clarity and appropriateness. Then based on the feedback from this poll test, the initially proposed questions were revised and finally measurement items listed in Table 1 were confirmed.

### 2.3. Stage 3: Data Collection and Cleaning

The platform we used to conduct the online survey is WJX (Changsha Ranxing Information Technology Co., Ltd. Changsha, China) which is considered as the most influential professional online survey website in China. Following common practices [31], the self-administered online survey approach was adopted to collect the empirical data. Using the paid random sampling service provided by WJX, survey links were randomly disseminated via popular social media platforms (such as WeChat, Weibo, and QQ) in the targeted provinces mentioned above.

To ensure the validity of the data, each IP address only has one chance to fill out the survey. Two survey waves were conducted on 9–11 March 2020 (the first stage, i.e., at the peak of the pandemic) and 25–30 March (the second stage, i.e., the period when China had removed the public health restrictions step by step), respectively. Data cleaning was conducted in three steps [32]: (1) excluding responses with apparent mistakes; (2) excluding responses containing a large proportion answers with the same numerical values; (3) excluding responses containing a large proportion of unanswered questions. Finally, a total of 697 (697/789, 88.3% response rate) and 1358 (1358/1547, 87.8%response rate) valid responses were collected in the first and second waves of survey, respectively. On average, the participants ranged in age from 18 to over 60. About 50% of the participants were female and 45.5% of participants had a bachelor’s degree.

### 2.4. Stage 4: Data Validity

Following Bhattacherjee [33], this study tested its data validity through comparing the basic features of the data we collected and the true target population. It is plausible that our data can be deemed representative for the general population if the notable demographic features of the respondents involved in this study (i.e., who used the internet to complete the online survey) are generally similar to those of Chinese internet users at the aggregated level. To do so, we obtained information regarding the overall features of Chinese internet users from CNNIC [23] for such a comparison. In general, the internet penetration rate in China is 67%, which implies the feasibility of a nationwide online questionnaire survey. Also, the male/female ratio of our sample is approximately 1, which is similar to that of Chinese internet users (i.e., 51.0:49.0). Finally, with respect to the age distribution, the percentages of respondents aged 18–35 and 36–45 were 43.1% and 30.6% respectively, which were higher than those of other age groups. In comparison, the percentages of respondents aged under 18 and over 60 were only 6.4% and 2.7%, respectively. This pattern was again generally consistent with the age distribution structure of Chinese internet users, which shows an inverted “U” curve. Therefore, the quality of data used in this study can be confirmed.

### 2.5. Theoretical Foundation of Modelling

**Figure 1:**
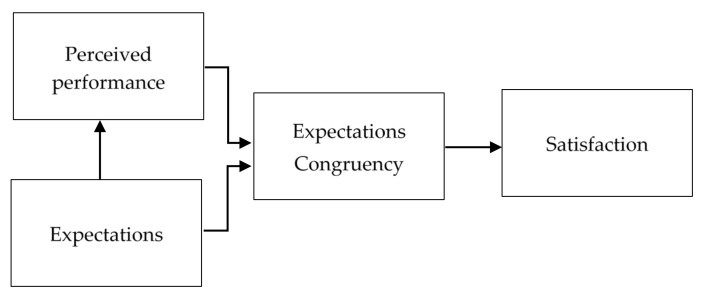
Conceptual Model of the Satisfaction Formation Process.

The model specification of this study follows the theory of customer satisfaction [34], where consumer satisfaction is affected by perceived behaviors, expectations, and expectations congruency. Specifically, with a positive expectation towards a subject or individual, a consumer would also have a positively perceived performance [35], which in turn determines an expectations congruency. If a performance is consistent with a consumer’s expectation, then the associated expectations congruency is likely to lead to a positive satisfaction situation. In other words, when perceived behavior can meet the requirement of an expected behavior, a consumer is likely to have the feeling of expectation consistency and then be satisfied (see Figure 1).

Along this line of thought, this study uses the thoery of conusmer satisfication as the theoretical foundation to construct the empirical model. The unpredicted presence of COVID-19 is a natural intervention between a consumer’s expectation and perceived performance, thus this scenario is appropriate to explore a difference in expectations congruency. Specifically, our model specified two situations: first, during the pandemic, a consumer’s perception and habits was affected by lockdown policies. It would be a difference between her/his expectation towards possible consequences brought by lockdown polices (e.g., lifestyles, shopping habits, etc.) and actual performances of these aspects this consumer perceived; second, after the pandemic, an expectation towards possible outcomes brought by unseal policies and actual perceived performances of these aspects. Therefore, our empirical model reveals either: (1) how the level of satisfaction is affected by the degree of expectations congruency on average; or (2) how a change of expectations congruency degree in wave2 compared to wave1 would affects the degree of satisfaction using a dummy variables method.

### 2.6. Analytical Models

In response to RQ1 and RQ2, this study uses both the method of comparative analysis and econometrics to deliver visual and robust results. First, given the nature of studies, many questions are not magnitude-based and thus cannot be measured by the Likert Scale approach. Even though it is plausible to encode these string variables to be numeric, it is difficult to compare attached qualitative information using traditional statistical approaches such as one-way ANOVAs [19]. Therefore, for exploring “the change of effect” but not “the difference of effect”, this study initially adopts the conventional descriptive approach (i.e., *t*-test). As the sampling method follows the principle of randomization, comparing these results between waves 1 and 2, to a great extent, provides meaningful information regarding how the socio-economic statue of the Chinese population changes during and after the pandemic.

Next, following the theory of consumer satisfaction, we can explicitly define the attitude towards public policy implementations as a specific proxy of satisfaction, and interviewees’ self-evaluations regarding income, expenditure, style, food price, food quality, education quality, food deliver method, etc. as perceived performances in response to expectations of consumers. Incorporating dummy variables can further distinguish the effects of such an expectations congruency between waves 1 and 2; this is beyond the scope of most of present studies that only focus on association analysis [18]. Specifically, we develop a LSDV [36] (Least Square Dummy Variable) model as below:

satisfaction_i_ = α + β_1_income_i_ + β_2_expenditure_i_ + β_3_style_i_ + β_4_foodprice_i_ + β_5_foodquality_i_ + β_6_educationquality_i_ + β_7_fooddeliver_i_ + β_8_ interactive term_i_ + controls_i_ + dummies + ε_i_ (1)

Compared to an OLS model that assumes a constant intercept through different data layers or the fixed effects model that requests observations remains the same in a temporal order, a LSDV model allows intercepts to vary in different sub-samples. Therefore, it is an effective technique to distinguish the effects in expectations congruency on satisfaction in the data of wave1 and 2 in this study; the input of dummy variables does not control the effects of each observations, instead, only the effect of survey wave is considered.

In Equation (1), the dependent variable is the degree of satisfaction towards the implementation of public health policies in general and the core independent variables include income change (expected income), expenditure (consumption structure change), style (how to purchase daily supplies), food price (price of necessities), food quality (a degree of satisfaction towards food quality), education quality (a degree of satisfaction towards online primary and middle school course or a degree of satisfaction towards online adult education) and food delivery (if choose a take-away food service). The control variables refer to demographic features of interviewees such as occupation, gender, age, educational attainment, etc. As mentioned above, our model incorporates the interactive term between the dummy variable of survey waves (i.e., wave1 = 0 and wave2 = 1) and each core independent variable respectively. Therefore, the incremental or decremental effect of a core independent variable on the degree of satisfaction after the pandemic can be captured compared to those of during the pandemic.

## 3. Results

### 3.1. Descriptive Analysis: From a Comparative Perspective

A T-test is employed as a complementary measure for confirming whether the difference by comparing the results based on the waves 1 and 2 surveys can be attributed to the influence of external factors such as the lockdown policy. If the hypothesis of equal variance is rejected, it means that the difference between two samples is very less likely caused by the issue of research design including selection bias.

#### 3.1.1. Income and Expenditure Level

**Figure 2:**
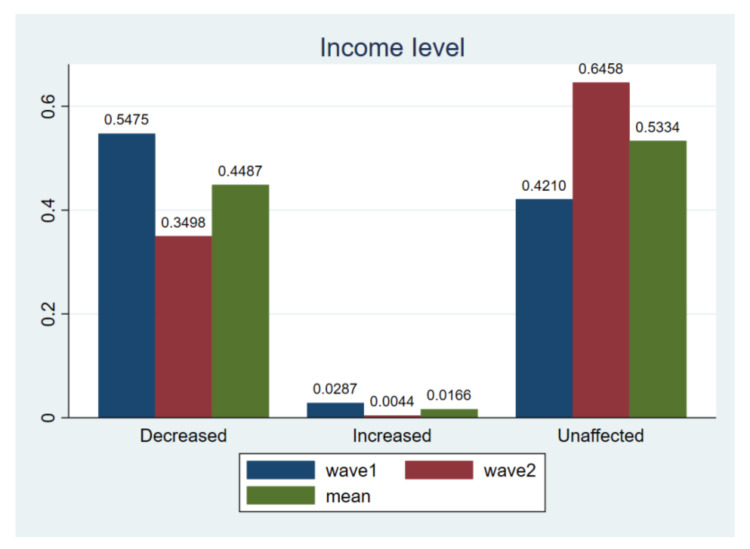
Comparing income level. Notes: *t*-test for H_0_: equal variance assumed: *t* value = 8.5551, Pr (|T| > |t|) = 0.0000; wave1 vs. wave2.

As shown in Figure 2, the proportion of people with a perception of income decease was 54.75% in wave1 compared to that of 34.98% in wave2. However, the proportion of people with a perception of income unaffected increased from 42.1% to 64.58%. In addition, respondents with a decreased income expectation of more than 3000 Chinese yuan accounted for 20% of the total number, and from the perspective of occupational characteristics, private owners, private enterprises and freelancers believed that their income levels were most negatively affected by COVID-19. This finding is consistent with Bodas [37] and Sandeep [38] who argued for a relatively short-term impact of the pandemic on people’s expected income.

**Figure 3:**
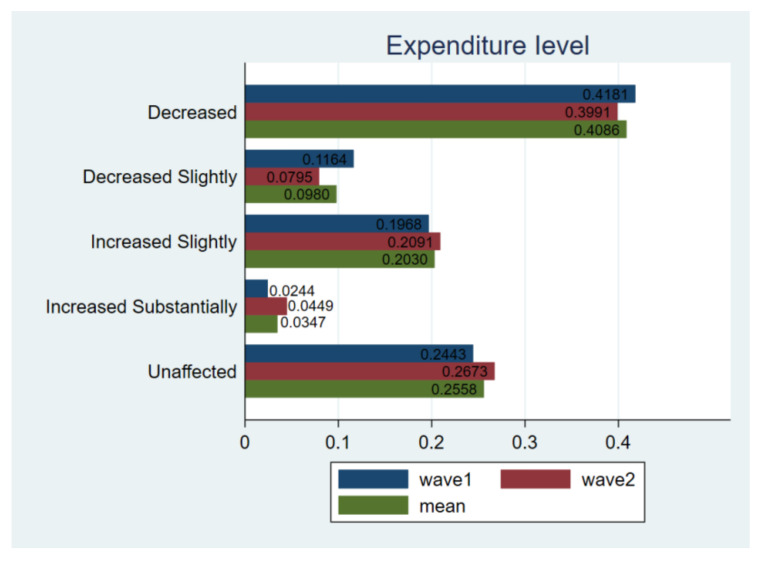
Comparing expenditure level. Notes: *t*-test for H_0_: equal variance assumed: *t* value = −2.1274, Pr (|T| > |t|) = 0.0335; wave1 vs. wave2.

As far as expenditure level is concerned, the proportion of respondents who thought their expenditures increased or unchanged became larger in wave2 (Figure 3), indicating that consumer confidence was gradually restoring with the pause of a lockdown policy. In detail, 23.7% of the respondents indicated that their total expenditure had increased, 56.7% of respondents’ food expenditure increased and 15.04% of respondents’ non-food expenditure increased.

**Figure 4:**
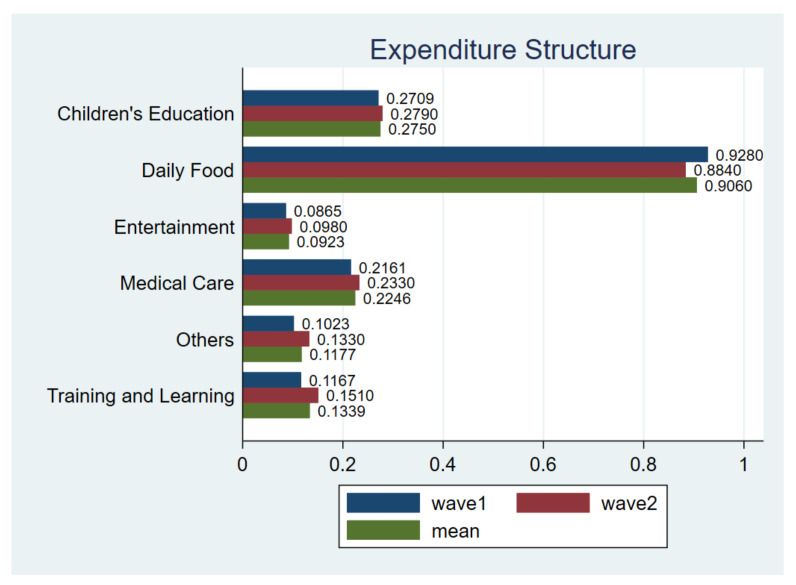
Expenditure structure. Notes: *t*-test for H_0_: equal variance assumed: *t* value = −1.1917, Pr (|T| > |t|) = 0.2335; wave1 vs. wave2.

From the perspective of expenditure structure (Figure 4), it is evident that the largest part of respondents’ spending was related to necessity, followed by children’s education and medical care, respectively. However, during the pandemic, respondents were less likely to focus on entertainment, which only accounts for 8.54% of the total sample. In the second wave of survey, such an expenditure structure was generally unchanged, where the ratio of spending on necessity decreased and the spending on the rest of categories slightly increased. This result suggests even though the national economy and social life gradually get back on track, consumers were still cautious about the pandemic situation in future.

#### 3.1.2. Purchase Method

**Figure 5:**
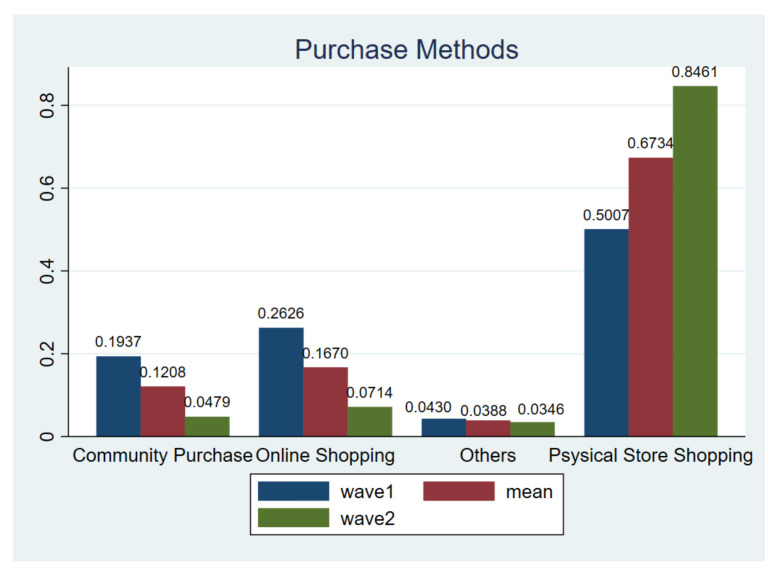
Purchase methods. Notes: *t*-test for H_0_: equal variance assumed: *t* value = −2.8744, Pr (|T| > |t|) = 0.0059; wave1 vs. wave2.

The finding indicates that shopping in store is still the main purchase method for respondents, showing a figure of 50.07% in wave1 and 84.61% in wave2. This finding is similar to Górnicka [39] based on the survey in Poland. In comparison, online shopping and community group purchases appears to be very popular in wave1, but as long as the national pandemic was efficiently controlled, most of respondents still prefer traditional shopping habits (Figure 5). This finding can be deemed realistic for two reasons: first, in many non-metropolitan areas in China, an e-commerce network is not fully established thus purchasing necessities in stores and through community group purchases are the only two viable options for a large proportion of Chinese residents; second, many local governments even prohibited the customer logistics operations during the pandemic outbreak. Therefore, it is not surprising that 84.61% of respondents returned to their original way of buying in the second wave of survey, while use frequency of both online-shopping and community group purchases decreased significantly.

#### 3.1.3. Online Education

During the pandemic, many provinces in China postponed the start date of school [40]. The education department responded quickly and proposed a non-stop school plan to encourage teachers and students to carry out online education activities [41]. A comparison was made towards the use of online classroom for primary, secondary schools and adults, and the associated degree of satisfaction.

**Figure 6:**
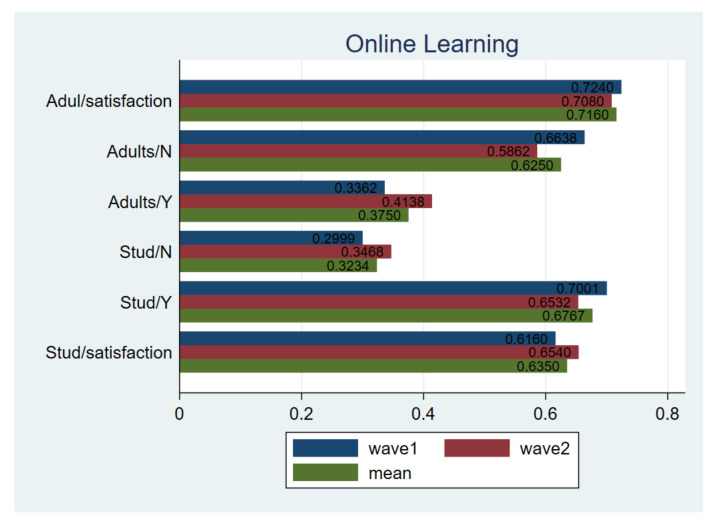
Comparing online learning. Notes: *t*-test for H_0_: equal variance assumed: *t* value = −2.1440, Pr (|T| > |t|) = 0.0321; adults online: *t*-test for H_0_: equal variance assumed: *t* value = 2.9681, Pr (|T| > |t|) = 0.0030; children satisfaction: *t*-test for H_0_: equal variance assumed: *t* value= −3.1664, Pr (|T| > |t|) = 0.0016; adults satisfaction: *t*-test for H_0_: equal variance assumed: *t* value= 0.9918, Pr (|T| > |t|) = 0.3216; wave1 vs. wave2.

Overall, the coverage of online education reached almost 100% during the pandemic, but the feedback of online courses was not accordingly high. Specifically, only 61.60% primary and secondary school students claimed that the quality of online course was satisfactory and this figure is 72.4% for adults (Figure 6). A similar pattern was still captured in wave2, as only about 65% children and 70% adults were satisfied with online courses. The T-test for equal variance assumed further revealed that there is no substantial difference regarding respondents’ satisfaction during and after the pandemic.

#### 3.1.4. The Preference of Take-Away Food

**Figure 7:**
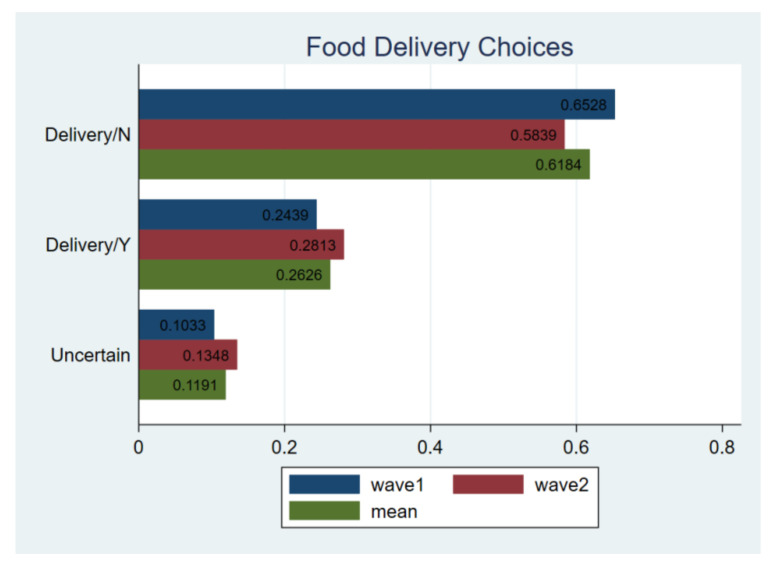
Comparing delivery choices. Notes: *t*-test for H_0_: equal variance assumed: *t* value = 0.2088, Pr (|T| > |t|) = 0.8346; wave1 vs. wave2.

Overall, respondents were concerned about the safety of take-away food. As Figure 7 shows, about 65% respondents preferred to eat at home in wave1. Such a ratio was slightly decreased in wave2, implying that consumer’s confidence was slowly restoring.

**Figure 8:**
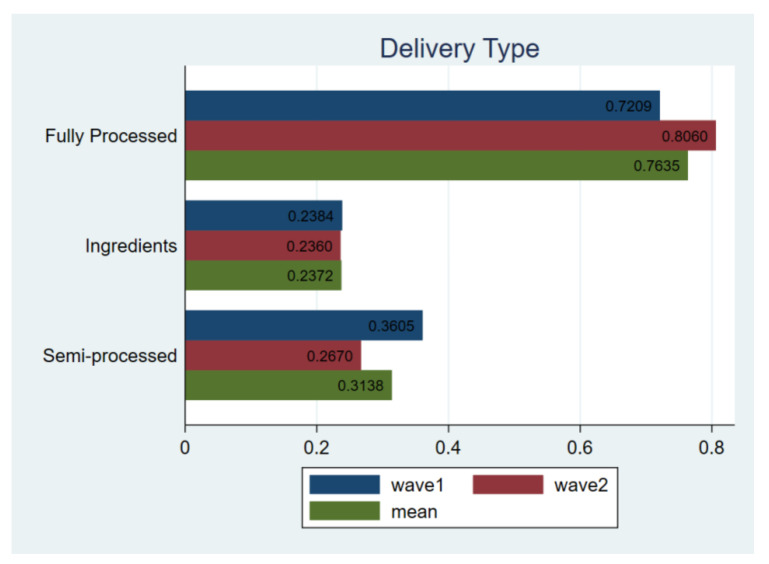
Comparing delivery types. Notes: *t*-test for H_0_: equal variance assumed: *t* value = −0.7620, Pr (|T| > |t|) = 0.4462; wave1 vs. wave2.

Next, a further detailed analysis shows that 24.39% of respondents still chose food delivery services, and 13.48% were not sure in wave2, suggesting that the willingness to purchase deliver food increased after the outbreak. 76.36% of respondents would choose fully processed food, and 31.83% would choose semi-processed food (Figure 8). Compared to the status in wave1, it is believed that although the pandemic has a limited impact on catering consumption, processed food was still the best choice to reassure respondents. The T-test also confirmed this pattern, as there is no substantial difference between the results in wave1 and 2.

#### 3.1.5. Price and Quality

**Figure 9:**
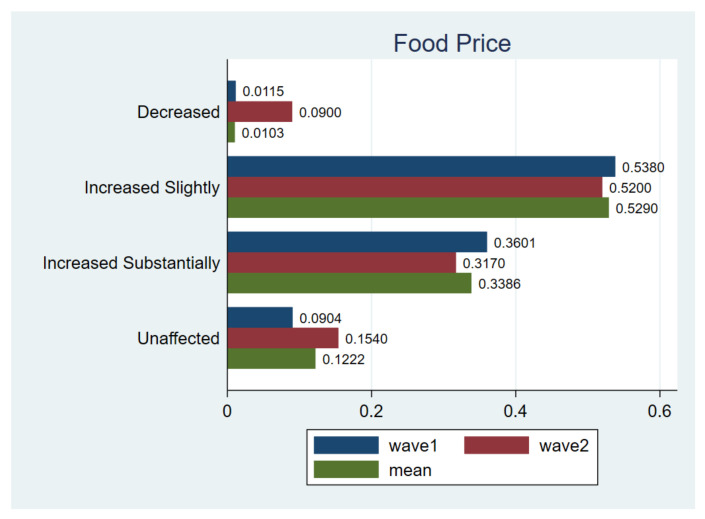
Comparing food price. Notes: *t*-test for H_0_: equal variance assumed: *t* value= −3.1645, Pr (|T| > |t|) = 0.0016; wave1 vs. wave2.

The change pattern is much more dramatic for food prices compared to other categories. About 86% of respondents believed that food price has risen while on average only 1.03% respondents thought they had fallen (Figure 9). It can be found that the implementation of emergent public health policies has a certain impact on the food price level, and the food expenditure inevitably increased as prices rose. In the second wave of the survey, even though fewer respondents thought food prices had increased, the ratio was still maintained at a substantial level.

**Figure 10:**
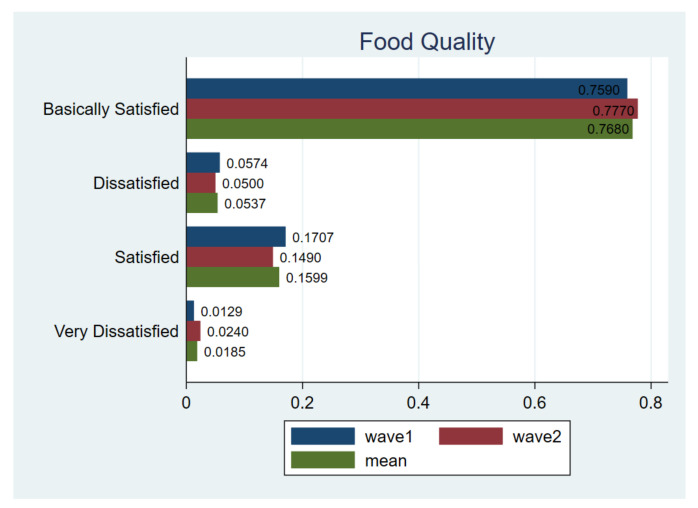
Comparing food quality. Notes: *t*-test for H_0_: equal variance assumed: *t* value = −14.8343, Pr (|T| > |t|) = 0.0000; wave1 vs. wave2.

Finally, as can be seen in Figure 10, the majority of respondents were generally satisfied with the quality of food. On average, about 93% of respondents were satisfied, among which 15.99% were satisfied and 76.8% were basically satisfied. It is worth mentioning that the largest T-test value was observed in this category, suggesting a dramatic change in respondents’ answer structure in wave1 and 2.

In summary, people’s life patterns, to a certain degree, were affected by the implementation of emergent public health policies such as a lockdown. However, the degree of this impact varies among different indicators; it appears that the most fundamental aspects of life including food quality and price and people’ s income and expenditure levels have a high probability of being negatively affected. In comparison, people’s choices related to lifestyle and habits, including expenditure structure, online learning, take-away food etc. only underwent slight changes during and after the pandemic. These findings imply that the socio-economic cost of public health policies towards COVID-19 seems to be very specific and multifaceted.

### 3.2. Econometric Analysis

Summary statistics are provided in Table 2. It appears that a significant heterogeneity among different variables can be observed. For instance, the average ranking of food prices is only 1.819, compared to that of food quality with a much higher value of 3.443. The mean value of income is only 1.429, which implies a general decline in income. In comparison, people’s satisfaction towards public health policies can be deemed positive, as the mean value of the satisfaction level is 4.345.

The above descriptive analysis, to a certain extent, shows that the changing life patterns of Chinese residents are evident during and after the pandemic. In this section, the casual relationship between the proxy of public health policy efficiency and changing patterns of people’s life choices is examined further. As most of the variables involved in this study are categorical, a LSDV model facilitates the interpretation of regression results. If only the independent effect of the socio-economic aspects involved in this study is concerned, the column 1, Table 3 shows that the status of respondents’ income level, purchase method, food price and survey period do not have significant impacts on the degree of satisfaction for respondents. In comparison, respondents who stated the total expenditure was “slightly decreased” in relative to the answer of “decrease” has a positive effect on the degree of satisfaction. This finding implicates that the determinants of attitudes towards public health polices is less likely to be identified at an aggregated level, instead, only a specific perception range would have an impact. Similarly, this pattern can be also found in the context of food quality, online learning and choosing take-away food. For instance, respondents with a more positive evaluation towards food quality led to a higher level of satisfaction, and with an answer “no” to food delivery has a more positive effect compared to those who said “yes”. These findings show that socio-economic cost of changing life patterns is evidently related to people’s overall attitudes towards a public health policy.

However, an aggregated analysis, without further considering how people’s changing life patterns vary during and after the pandemic, fails to depict the role of policy-making, thus the interactive terms between survey period and each socio-economic indicator were incorporated. From Column 2 to Column 9, the sign and significance of each independent variable is generally similar to Column 1, suggesting our results, to a certain degree, are robust across different model specifications (i.e., with different interactive terms). It appears that only food price and quality have differential effects on the degree of satisfaction about the efficiency of public health policies in the second wave of survey compared to those of in wave1 i.e., the negative effect of respondents’ attitudes towards price increase on the degree of satisfaction decreased and the positive effect of respondents’ perception towards food quality on the degree of satisfaction increased in the second wave of survey. This is an important finding in showing that the implementation of a lockdown policy may not have a direct impact on lifestyle-related behaviors.

## 4. Discussion

Inevitably, the sudden outbreak of COVID-19 has disturbed the normal pace of life for everybody. Therefore, it is not surprised that 44.87% of the respondents reported their income levels has decreased, a finding is in line with many previous studies showing that wage earnings were reduced [42,43,44] during the pandemic, and the loss of income has a negative impact on people’s purchasing power. This could explain another finding that 50.66% of the respondents’ expenditure levels have also decreased. However, respondents’ consumption structure and purchase method did not appear to be significantly impacted, as food, education and medical care are still the top three preferences, and respondents still chose to shop in stores; the survey shows that about 67.34% of respondents selected shopping in stores in the first wave, and the number increased up to 84.61% in the second wave. In addition, respondents’ preferences regarding food delivery choice, food delivery type and online learning also only displyed slight changes during and after the pandemic, which are however statistically insignificant. This finding is similar to a number of present studies. For instance, Cavallo et al. [45] believed that an embodiment of consumer’s purchase resilience plays a larger role even a consumer’s lifestyle is temporally affected by a lockdown policy.

The econometric analysis further revealed that quality and price are the only two indicators that have differential impacts on the degree of people’s satisfaction towards a public health policy implication. This finding again, from a different perspective, proves that only fundamental living needs would alter people’s attitudes and such an effect is likely to be different during and after the pandemic. The implementation of lock-down policy may initially lead to a deterioration of food quality and an increase of price. However, the reaction of consumers rapidly changed and became more positive at the second stage, where the restrictions were gradually loosened. This finding, on the other hand, implies that government’s responding policy towards the basic quality of people’s lives was efficient. In comparison, many existing studies also provided similar or contradictory patterns about the impact of lock-down policies on essential aspects of people’s lives. For instance, food quality and security was found to be generally decreased in Kenya and Uganda during the pandemic [46]. Lucile stated that the decrease of food quality is associated with food choice motive changes in France [47]. Jia et al. [48] revealed that being remained at home for the purpose of social distancing increased a demand for food delivery services due to the caution to avoid human-to-human contact [49]. Nevertheless, this literature thread is mainly based on a “before and during” scope and cannot further discuss how people’s consumption behaviors would change towards COVID-19 in a “during and after” scenario. This is caused by the fact that the spread of virus has yet efficiently controlled in most of the countries, thus a “during and after” comparison is difficult to conduct.

Considering all the above, the present study provides an interesting finding compared to existing studies, that is, Chinese consumers did not appear to dramatically change their life choices forged during the period of home quarantine. The reason is twofold: first, due to the fear of reinfection [50], the existing environmental settings did not produce much stronger risk-reducing and purchase-intention effects that make consumers to change their “new” habits [51]; on the other hand, this finding may highlight that the effect of safety signals such as shopping online, choosing take-away food, diminishes alongside other types of signals [52]. These evidences highlight the complexity of human behaviors and reactions towards major disasters.

## 5. Conclusions, Implications and Research Limitations

Comparing the status during and after the pandemic, this study proposes two analytical frameworks to explore how Chinese residents’ life patterns alter in response to a catastrophic event and associated public health policies. Based on a rigorous sampling method, the initial descriptive analysis reveals that respondents’ lifestyles did not significantly change such as purchasing methods, consumption patterns, and education, while respondents were more likely to have a differential “attitude” towards price and quality of food during and after the pandemic. An econometric analysis further confirms this pattern, as only quality and price of food had a significant effect on the degree of satisfaction towards the implementation of public health policies. With this in mind, these findings, on one hand, implies that most of the basic indicators mentioned in this study failed to deliver a promising signal to consumers; and on the other hand, consumer’s hesitation indicates that the socio-economic cost of a lock down policy is considerably high.

This study has several implications for policy makers at both the macro and micro levels. At the micro level, if an implementation of lock-down policy is periodical in a post-pandemic world, policy makers have to thoroughly evaluate its associated socio-economic costs and be prepared for a new habit formation tendency, in addition to a general decline in income. Consumers’ sensitivities to price and quality of food provide useful insights to assist local regulators in their recover efforts following the implementation of lockdown policy. Among various policy options, focusing on price and quality control appears to be of primary importance. Particularly in the case where many Chinese consumers have no choices but to rely on physical store shopping, high-quality service/good providers need to enhance or reveal quality as a safety signal to consumers while for low-quality service/good providers, it is better to take a non-signaling strategy first such as physical cleaning actions or just improving quality. In addition, the level of price delivers revenue-risking signal to consumers, which can effectively enhance consumers’ general confidences and purchase intentions. Therefore, a sound price-open and supervision system can also help stimulate people’s behavioral changes and public health policy satisfaction.

From a macro perspective, public health policy makers shall notice that the socio-economic cost of a public health policy such as home guarantee is substantial and durable, in addition to direct mental and physical damages from the virus. Therefore, it may be ideal for the Chinese government to adopt more efficient and less costly public health policies to restore the confidence of consumers and markets in general. For instance, using the method of grid management, measures towards virus prophylaxis and treatment can be retained in limited areas, while people’s life is not affected elsewhere. However, taking advantage of perpetual habit and perception changes as an opportunity, enterprises could also just follow these new trends and develop new products and services with features that fit well with the requirements from newly emerged consumer segments. This attempt requires an effective coordination among different policy areas.

This study, however, has certain limitations. First, due to constraints caused by COVID-19 in March, in the data collection process is was difficult to observe the same interviewees over time, thus a panel analysis which could deliver an accurate estimation from the perspective of within-group variations was not feasible. Second, the current analytical framework cannot further reveal if respondents’ life choices would change in an extended period of lock down. Third, the questionnaire design was not very sophisticated in giving a full consideration that respondents were in no mood to answer a large number of questions when COVID-19 reached its peak in March, 2020, thus further research may propose more socio-economic variables to complement the existing findings. Finally, it is worth mentioning that the findings of this study are context-based. Consumers in different cultural backgrounds may have different perceptual and behavioral reactions to public health policies implemented during and after the pandemic. Therefore, the generalizability of the results obtained from this study needs to be verified for other cultural and institutional contexts.

## Figures and Tables

**Table 1:** Questionnaire design and basic statistics.

Perspectives	Variable Definitions	Measurement Scale (Corresponding to Variable Definitions)	Statistics
Demographic feature	Gender, N (%)		
	Male	1	Wave1 = 332 (47.63%); Wave2 = 696 (51.25%);
	Female	2	Wave1 = 365 (52.37%); Wave2 = 662 (48.75%);
	Age, N (%)		
	Under 18	1	Wave1 = 59 (8.46%); Wave2 = 73 (5.38%);
	18–35	2	Wave1 = 270 (38.74%); Wave2 = 617 (45.51%);
	36–45	3	Wave1 = 211 (30.27%); Wave2 = 419 (30.85%);
	46–60	4	Wave1 = 135 (19.37%); Wave2 = 216 (15.91%);
	60+	5	Wave1 = 22 (3.16%); Wave2 = 33 (2.43%);
	Educational attainment, N (%)		
	Below high school	1	Wave1 = 10 (1.43%); Wave2 = 89 (6.55%);
	High school/technical secondary school	2	Wave1 = 85 (12.2%); Wave2 = 129 (9.50%);
	Junior college	3	Wave1 = 51 (7.32%); Wave2 = 198 (14.58%);
	Undergraduates	4	Wave1 = 290 (41.61%); Wave2 = 691 (50.88%);
	Postgraduates and above	5	Wave1 = 261 (37.45%); Wave2 = 251 (19.22%);
	Occupation, N (%)		
	Government employee	1	Wave1 = 83 (11.91%); Wave2 = 166 (12.22%);
	Medical/nursing professional	2	Wave1 = 4 (0.57%); Wave2 = 28 (2.06%);
	Teaching-related professional	3	Wave1 = 246 (35.29%); Wave2 = 255 (18.78%);
	Private business owner	4	Wave1 = 13 (1.87%); Wave2 = 45 (3.31%);
	Employee at national companies	5	Wave1 = 76 (10.9%); Wave2 = 125 (9.20%);
	Employee in foreign companies	7	Wave1 = 9 (1.29%); Wave2 = 14 (1.04%);
	Employee in private companies	8	Wave1 = 106 (15.21%); Wave2 = 162 (11.93%);
	Freelancer	9	Wave1 = 32 (4.59%); Wave2 = 103 (7.58%);
	Student	10	Wave1 = 89 (12.77%); Wave2 = 368 (27.1%);
	Farmer	11	Wave1 = 9 (1.29%); Wave2 = 16 (1.18%);
	Unemployed	12	Wave1 = 12 (1.72%); Wave2 = 29 (2.14%);
	Retiree	13	Wave1 = 11 (1.58%); Wave2 = 28 (2.06%);
	Others	14	Wave1= 7 (1%); Wave2 = 19 (1.40%);
	Expected income, N (%)		
	Unchanged	1	Wave1 = 366 (52.49%); Wave2 = 877 (64.58%);
	Decrease	2	Wave1 = 247 (35.44%); Wave2 = 475 (35.00%);
	Increase	3	Wave1 = 84 (12.05%); Wave2 = 6 (0.44%);
Income and consumption structure	The largest proportion of family expenditure, N (%)		
	Daily supplies	1	Wave1 = 644 (92.8%); Wave2 = 1200 (88.37%);
	Children’s education	2	Wave1 = 188 (27.09%); Wave2 = 379 (27.91%);
	Entertainment	3	Wave1 = 60 (8.65%); Wave2 = 133 (9.80%);
	Study and re-skilling	5	Wave1 = 81 (11.67%); Wave2 = 205 (15.10%);
	Health care	6	Wave1 = 150 (21.61%); Wave2 = 316 (23.27%);
	Others	7	Wave1 = 71 (10.23%); Wave2 = 180 (13.25%);
	Consumption structure change (1 significantly decreased—5 significantly increased)		
	Total expenditure	1	Wave1:Mean = 2.29 SD = 1.26 Wave2:Mean = 2.42 SD = 1.32
	Food expenditure	2	Wave1:Mean = 3.38 SD = 1.23 Wave2:Mean = 3.35 SD = 1.18
	Non-food expenditure	3	Wave1:Mean = 2.15 SD = 1.18 Wave2:Mean = 2.20 SD = 1.18
Lifestyle	How to purchase daily supplies during the pandemic, N (%)		
	Internet	1	Wave1 = 83 (26.26%); Wave2 = 366 (26.95%);
	Physical store	2	Wave1 = 552 (50.07%); Wave2 = 766 (56.40%);
	Community group purchase	3	Wave1 = 46 (19.37%); Wave2 = 185 (13.62%);
	Others	5	Wave1 = 16 (4.3%); Wave2 = 71 (5.23%);
	Price of necessities (1 significantly decreased—5 significantly increased)	1–5	Wave1:Mean = 1.75 SD = 0.66 Wave2:Mean = 1.85 SD = 0.70
	Degree of satisfaction towards food quality (1 significantly decreased—5 significantly increased)	1–5	Wave1:Mean = 3.09 SD = 0.52 Wave2:Mean = 3.63 SD = 0.88
	If choose online primary and middle school course, N (%)		
	Yes	1	Wave1 = 488 (70.01%); Wave2 = 887 (65.32%);
	No	2	Wave1 = 209 (29.99%); Wave2 = 471 (34.68%);
	If choose online adult course, N (%)		
	Yes	1	Wave1 = 236 (33.62%); Wave2 = 562 (41.38%);
	No	2	Wave1 = 465 (66.38%); Wave2 = 796 (58.62%);
	Degree of satisfaction towards online primary and middle school course (1 significantly decreased—5 significantly increased)	1–5	Wave1:Mean = 3.1 SD = 1.17 Wave2:Mean = 3.28 SD = 1.03
	Degree of satisfaction towards online adult education (1 significantly decreased—5 significantly increased)	1–5	Wave1:Mean = 3.62 SD = 1.04 Wave2:Mean = 3.54 SD = 0.95
	If choose take-away food service, N (%)		
	Yes	1	Wave1 = 170 (24.39%); Wave2 = 382 (28.13%);
	No	2	Wave1= 455 (65.28%); Wave2 = 793 (58.39%);
	Uncertain	3	Wave1= 72 (10.33%); Wave2 = 183 (13.48%);
	The preference of take-away food, N (%)		
	Processed	1	Wave1 = 124 (72.09%); Wave2 = 308 (80.62%);
	Semi-processed	2	Wave1 = 62 (36.05%); Wave2 = 102 (26.70%);
	Raw materials	3	Wave1 = 41 (23.84%); Wave2 = 90 (23.57%);
Overall attitude	Attitude towards the efficiency of public health policies during the pandemic (1 strongly dissatisfied—5 strongly satisfied)	1–5	Wave1:Mean = 4.16 SD = 0.96 Wave2:Mean = 4.44 SD = 0.84

**Table 2:** Summary statistics.

	Mean	Max	Min	Standard Deviation
Satisfaction	4.345	5	1	0.888
Gender	1.563	2	1	0.496
Age	2.664	5	1	0.924
education	3.775	5	1	1.081
Occupation	5.726	13	1	3.162
Family	2.579	5	1	1.017
Income	1.429	3	1	0.531
Purchase method	2.001	4	1	0.777
Food price	1.819	4	1	0.686
Food quality	3.443	5	1	0.819
Adult education	3.556	5	1	0.969
Children education	3.202	5	1	1.092
Food delivery	1.855	3	1	0.609

**Table 3:** Independent effect of core variables on satisfaction.

	Basic	Income	Method	Price	Expenditure	Quality	Adult Learning	Children Learning	Food
Income (reference group: not significantly)									
Decrease	−0.125	−0.257	−0.122	−0.129	−0.144	−0.119	−0.120	−0.127	−0.119
	(0.0726)	(0.141)	(0.0728)	(0.0725)	(0.0734)	(0.0725)	(0.0732)	(0.0732)	(0.0727)
Increase	0.637	0.763	0.651	0.652	0.563	0.446	0.681	0.594	0.616
	(0.384)	(0.526)	(0.386)	(0.382)	(0.392)	(0.395)	(0.389)	(0.391)	(0.385)
Purchase method: (reference group: internet)									
Instore	0.0694	0.0698	0.0490	0.0610	0.0556	0.0648	0.0703	0.0695	0.0667
	(0.0733)	(0.0733)	(0.157)	(0.0731)	(0.157)	(0.0732)	(0.0734)	(0.0735)	(0.0733)
Group	0.0719	0.0763	−0.0560	0.0858	−0.0794	0.0790	0.0821	0.0764	0.0594
	(0.0997)	(0.0998)	(0.207)	(0.0993)	(0.207)	(0.0997)	(0.100)	(0.100)	(0.100)
Other	−0.0288	−0.0437	0.280	−0.0256	0.215	−0.0235	0.00465	−0.0546	−0.0271
	(0.171)	(0.171)	(0.382)	(0.170)	(0.381)	(0.170)	(0.172)	(0.172)	(0.171)
Expenditure level (reference group: decreased)									
Decreased slightly	0.286 *	0.294 *	0.290	0.287 *	0.286 *	0.308 *	0.275 *	0.295 *	0.286 *
	(0.120)	(0.120)	(0.215)	(0.120)	(0.120)	(0.120)	(0.120)	(0.121)	(0.120)
Unchanged	−0.0530	−0.0468	0.0348	−0.0499	−0.0505	−0.0397	−0.0689	−0.0484	−0.0493
	(0.0781)	(0.0782)	(0.177)	(0.0783)	(0.0780)	(0.0784)	(0.0788)	(0.0785)	(0.0781)
Increased	0.00994	0.0103	0.0652	0.00708	0.00966	0.0274	0.00492	0.0123	0.00824
	(0.0851)	(0.0852)	(0.188)	(0.0854)	(0.0849)	(0.0855)	(0.0854)	(0.0857)	(0.0854)
Increased substantially	0.226	0.254	0.215	0.226	0.193	0.261	0.231	0.209	0.214
	(0.160)	(0.165)	(0.163)	(0.160)	(0.160)	(0.160)	(0.160)	(0.161)	(0.160)
Food price (reference group: significantly increase)									
Increase	−0.0339	−0.0329	−0.0341	0.229	−0.0105	−0.0306	−0.0459	−0.0385	−0.0497
	(0.0752)	(0.0753)	(0.0755)	(0.149)	(0.0761)	(0.0751)	(0.0758)	(0.0758)	(0.0758)
Unchanged	−0.116	−0.114	−0.114	0.133	−0.0790	−0.104	−0.120	−0.112	−0.123
	(0.109)	(0.110)	(0.110)	(0.280)	(0.110)	(0.110)	(0.110)	(0.110)	(0.110)
Decrease	0.330	0.332	0.342	1.675 *	0.407	0.196	0.236	0.390	0.366
	(0.334)	(0.334)	(0.336)	(0.748)	(0.335)	(0.340)	(0.340)	(0.337)	(0.334)
Food quality: (reference group: very unsatisfied)									
Unsatisfied	0.261	0.250	0.250	0.249	0.244	−0.227	0.194	0.285	0.296
	(0.273)	(0.273)	(0.274)	(0.274)	(0.273)	(0.423)	(0.276)	(0.275)	(0.274)
Natural	0.989 ***	0.992 ***	0.986 ***	1.027 ***	0.998 ***	−0.167	0.893 ***	1.051 ***	1.016 ***
	(0.241)	(0.242)	(0.242)	(0.241)	(0.241)	(0.181)	(0.248)	(0.246)	(0.242)
Satisfied	1.247 ***	1.251 ***	1.239 ***	1.287 ***	1.252 ***	omitted	1.155 ***	1.319 ***	1.265 ***
	(0.243)	(0.244)	(0.244)	(0.244)	(0.244)	omitted	(0.250)	(0.249)	(0.244)
Very satisfied	1.531 ***	1.542 ***	1.525 ***	1.571 ***	1.544 ***	1.533 ***	1.441 ***	1.611 ***	1.560 ***
	(0.257)	(0.258)	(0.257)	(0.257)	(0.257)	(0.256)	(0.264)	(0.264)	(0.257)
Adult online learning (reference group: very unsatisfied)									
Unsatisfied	0.148	0.146	0.142	0.196	0.144	0.164	−0.419	0.0665	0.156
	(0.217)	(0.218)	(0.218)	(0.218)	(0.219)	(0.217)	(0.415)	(0.230)	(0.217)
Natural	0.427 *	0.435 *	0.428 *	0.450 *	0.427 *	0.411	0.0579	0.348	0.447 *
	(0.210)	(0.211)	(0.210)	(0.210)	(0.210)	(0.209)	(0.366)	(0.218)	(0.210)
Satisfied	0.519 *	0.527 *	0.522 *	0.548 **	0.508 *	0.507 *	0.0199	0.444 *	0.528 *
	(0.210)	(0.211)	(0.211)	(0.210)	(0.211)	(0.210)	(0.353)	(0.219)	(0.210)
Very satisfied	0.627 **	0.635 **	0.623 **	0.660 **	0.611 **	0.620 **	0.197	0.547 *	0.630 **
	(0.223)	(0.224)	(0.224)	(0.223)	(0.224)	(0.222)	(0.378)	(0.230)	(0.223)
Children’s online learning (reference group: very unsatisfied)									
Unsatisfied	−0.424 *	−0.424 *	−0.439 *	−0.408 *	−0.409 *	−0.437 *	−0.343	−0.146	−0.436 *
	(0.178)	(0.179)	(0.179)	(0.178)	(0.180)	(0.178)	(0.185)	(0.373)	(0.179)
Natural	−0.155	−0.157	−0.167	−0.127	−0.137	−0.176	−0.0851	0.0572	−0.171
	(0.167)	(0.167)	(0.167)	(0.166)	(0.168)	(0.167)	(0.171)	(0.301)	(0.167)
Satisfied	−0.0327	−0.0442	−0.0506	−0.0146	−0.00664	−0.0594	0.0350	0.364	−0.0493
	(0.171)	(0.171)	(0.172)	(0.170)	(0.173)	(0.171)	(0.176)	(0.313)	(0.171)
Very satisfied	−0.0463	−0.0491	−0.0466	−0.0152	−0.000109	−0.0454	0.0343	0.277	−0.0538
	(0.196)	(0.197)	(0.197)	(0.196)	(0.198)	(0.196)	(0.202)	(0.327)	(0.196)
Take-away food (reference group: yes)									
No	0.160 *	0.159 *	0.155 *	0.152 *	0.142	0.162 *	0.163 *	0.164 *	0.145
	(0.0735)	(0.0735)	(0.0737)	(0.0733)	(0.0737)	(0.0733)	(0.0736)	(0.0739)	(0.175)
Not sure	0.254 *	0.258 *	0.244 *	0.265 *	0.212	0.243 *	0.239 *	0.275 *	−0.104
	(0.110)	(0.110)	(0.110)	(0.110)	(0.111)	(0.110)	(0.111)	(0.111)	(0.267)
Wave (reference group: wave1)									
wave2	0.0603	−0.0265	0.0374	0.300 *	0.0380	−1.103 ***	−0.546	0.415	0.0106
	(0.0888)	(0.122)	(0.140)	(0.139)	(0.140)	(0.290)	(0.381)	(0.306)	(0.169)
No interactive terms									
	Income * wave								
Decrease * wave2		0.174 (0.160)							
Increase * wave2		−0.329 (0.773)							
		Purchase method * wave							
Instore * wave2			0.0255 (0.175)						
Group * wave2			0.163 (0.234)						
Other * wave2			−0.377 (0.422)						
			Price * wave						
				−0.351 * (0.168)					
				−0.327 (0.299)					
				−1.697 * (0.829)					
				Expenditure * wave					
Decreased slightly					0.00385 (0.265)				
Unchanged					−0.109 (0.197)				
Increased					−0.0700 (0.212)				
Increased substantially					omitted				
					Quality * wave				
Unsatisfied * wave2						0.364 (0.510)			
Neutral * wave2						1.167 *** (0.306)			
Satisfied * wave2						1.273 *** (0.244)			
Very satisfied * wave2						omitted			
						Adult online learning * wave			
Unsatisfied * wave2							0.767 (0.470)		
Neutral * wave2							0.524 (0.412)		
Satisfied * wave2							0.684 (0.398)		
Very satisfied * wave2							0.584 (0.422)		
							Children online learning * wave		
Unsatisfied * wave2								−0.356 (0.408)	
Neutral * wave2								−0.279 (0.330)	
Satisfied * wave2								−0.509 (0.339)	
Very satisfied * wave2								−0.437 (0.365)	
								Take-away * wave	
No * wave2									0.0101 (0.191)
Not sure * wave2									0.433 (0.292)
Demographic features (controls)	Yes	Yes	Yes	Yes	Yes	Yes	Yes	Yes	Yes
Constant	2.572 ***	2.638 ***	2.590 ***	2.278 ***	2.412 ***	3.748 ***	3.056 ***	2.297 ***	2.560 ***
	(0.401)	(0.405)	(0.412)	(0.416)	(0.413)	(0.393)	(0.492)	(0.454)	(0.412)
*N*	525	525	525	525	525	525	525	525	525
*R* ^2^	0.387	0.389	0.389	0.397	0.402	0.392	0.392	0.391	0.391

Notes: dependent variable: the degree of satisfaction towards public health policies, robust standard errors in parentheses, * *p* < 0.10, ** *p* < 0.05, *** *p* < 0.01, demographic features include gender, age, educational attainment, and occupation which are not displayed in the table.

## Data Availability

The data presented in this study are available on request from the corresponding author. The data are not publicly available due to confidentiality of personal information.
